# Tissue Invasion by *Entamoeba histolytica:* Evidence of Genetic Selection and/or DNA Reorganization Events in Organ Tropism

**DOI:** 10.1371/journal.pntd.0000219

**Published:** 2008-04-09

**Authors:** Ibne Karim M. Ali, Shahram Solaymani-Mohammadi, Jasmine Akhter, Shantanu Roy, Chiara Gorrini, Adriana Calderaro, Sarah K. Parker, Rashidul Haque, William A. Petri, C. Graham Clark

**Affiliations:** 1 Department of Infectious and Tropical Diseases, London School of Hygiene & Tropical Medicine, London, United Kingdom; 2 Division of Infectious Diseases and International Health, University of Virginia Health System, Charlottesville, Virginia, United States of America; 3 Division of Intestinal and Genital Protozoal Diseases, Department of Medical Parasitology and Mycology, School of Public Health and Institute of Public Health Research, Tehran University of Medical Sciences, Tehran, Iran; 4 International Centre for Diarrhoeal Disease Research, Dhaka, Bangladesh; 5 Department of Medicine, University of Chicago, Chicago, Illinois, United States of America; 6 Department of Pathology and Laboratory Medicine, Section of Microbiology, University of Parma, Parma, Italy; 7 Department of Pediatrics, Division of Infectious Diseases, University of Colorado Health Sciences Center and The Children's Hospital, Aurora, Colorado, United States of America; Bose Institute, India

## Abstract

*Entamoeba histolytica* infection may have various clinical manifestations. Nine out of ten *E. histolytica* infections remain asymptomatic, while the remainder become invasive and cause disease. The most common form of invasive infection is amebic diarrhea and colitis, whereas the most common extra-intestinal disease is amebic liver abscess. The underlying reasons for the different outcomes are unclear, but a recent study has shown that the parasite genotype is a contributor. To investigate this link further we have examined the genotypes of *E. histolytica* in stool- and liver abscess-derived samples from the same patients. Analysis of all 18 paired samples (16 from Bangladesh, one from the United States of America, and one from Italy) revealed that the intestinal and liver abscess amebae are genetically distinct. The results suggest either that *E. histolytica* subpopulations in the same infection show varying organ tropism, or that a DNA reorganization event takes place prior to or during metastasis from intestine to liver.

## Author Summary

The parasite *Entamoeba histolytica* can cause serious disease by invading the lining of the large intestine and spreading to other organs of the body. However, most infected individuals never develop symptoms, and it is not clear what determines the different outcomes of infection. Factors that might be having an effect range from the immune response of the infected individual, to concurrent infections with other organisms or genetic differences among the parasites. In the present study we investigated the role of the latter by comparing parasites in the intestine with those that have invaded the liver of the same patient. In all 18 pairs of samples we could detect genetic differences among the parasites. Interpreting the findings is difficult, as we cannot distinguish at present between mutations that have occurred during tissue invasion and genetic diversity that was already present in the population of parasites in the intestine. However, our results strongly support a role for the parasite in determining the outcome of infection with *E. histolytica*.

## Introduction

*Entamoeba histolytica*, the causative agent of human amebiasis, is endemic in many tropical countries. It is well established that *E. histolytica* infections result in variable clinical outcomes. Most infections remain asymptomatic, some develop diarrhea and dysentery, and only a few develop extra-intestinal complications such as liver abscess. The parasite is estimated to be responsible for millions of cases of dysentery and liver abscess world-wide annually, resulting in up to 100,000 deaths [1],[2] Additionally, *E. histolytica*-associated dysentery is associated with malnutrition and lower cognitive test scores in infected children in Bangladesh, and thus may impair their proper development [3],[4].

What determines the outcome of an *E. histolytica* infection remains largely unknown although host genetics and parasite genotype likely play important roles [5],[6]. We recently investigated the association between the genotypes of parasites and the clinical outcome of infection (6) using Bangladeshi clinical samples and a 6-locus genotyping system based on tRNA-linked short tandem repeats (STRs) [7]. Our results indicated that there exists a population-level association between genotypes and outcome of infection, as the distribution of *E. histolytica* genotypes among asymptomatic, diarrhea/dysentery, and liver abscess patients showed significant differences. Surprisingly, genotypes present in liver abscess samples were only rarely encountered in asymptomatic individuals [6],[8].

The preceding work suggests that not all genotypes of *E. histolytica* have the same capacity to cause liver abscess and that differential selection among genotypes may occur in the intestine. To pursue this observation further, we needed to move from the population to the individual infection level and compare intestinal and invasive specimens from the same patient. In this report we investigate the genotypes of 18 paired stool and extraintestinal samples from individuals in 3 countries. In each case genetic differences could be detected between parasites in the intestinal and hepatic sites of infection.

## Materials and Methods

### Samples

16 amebic liver abscess (ALA) patients from various private and public hospitals and clinics in Dhaka, Bangladesh provided both liver abscess pus and stool specimens during 1998–99 [9] and 2006–2007. The Ethical Review Committee of the International Centre for Diarrhoeal Disease Research, Bangladesh (ICDDR,B), and the Human Investigation Committee of the University of Virginia reviewed and approved the design of the study under which these samples were obtained. Samples from a 16 month-old child in Colorado, USA, with a ruptured ALA, and stomach perforation, peritoneal, pleural and pericardial extension were also available. For simplicity these extra-intestinal samples will be referred to as deriving from ALA pus. These samples were obtained during differential diagnosis. The Colorado Multiple Institutional Review Board concluded that the additional analyses did not constitute ‘human research’ and thus did not require their approval. Liver abscess pus and stool specimens were available from one ALA patient in Parma, Italy (patient 2 in reference [10]). These had been submitted for routine diagnosis and no approval by the local review committee was required.

### Isolation of DNA

DNA was isolated directly from the stool samples or ALA pus samples (i.e. no prior culturing steps were involved) by using different conventional methods. The CTAB DNA extraction method described previously [7] was employed for the stool and pus specimens from Bangladesh. The DNA from the Italian samples was isolated using the HighPure PCR Template preparation kit (Roche Diagnostics, Germany). DNA from the USA samples was isolated using the QIAamp DNA Stool Mini Kit (Qiagen, Germany).

### Polymerase Chain Reaction (PCR)

In order to determine the parasite genotypes we used a species-specific PCR amplification method [7]. This genotyping method has demonstrated excellent discriminatory power in identifying individual variants of *E. histolytica* by amplifying 6 highly repetitive regions containing STRs flanked by tRNA genes. In order to obtain amplification from some samples we used “tRNA primers” in the first PCR followed by nested PCR using internally located *E. histolytica* species-specific primers in the second [7]. All the primer sequences and thermocycler settings were as previously described [7]. PCR was performed using either standard BioLine Taq (UK and USA) or high-fidelity Sahara DNA polymerase (BioLine, USA) with 1.5 mM MgCl_2_ (BioLine, USA). All PCR products were separated in 1.5% agarose gels in 1× Tris-Borate-EDTA buffer. For the most part, the size marker used was a 100 bp ladder.

### Cloning and sequencing of amplified products

Some of the Bangladeshi and Italian PCR amplified products were sequenced directly while the remainder of Bangladeshi amplified products and the USA amplified products were cloned using the TOPO TA vector system and One Shot Chemically Competent Cells (both from Invitrogen) according to the manufacturer's instructions. Plasmid DNA was isolated using the QIAprep Spin Mini Kit (Qiagen). Sequences were obtained using the ABI Prism® BigDye^TM^ terminator cycle sequencing ready reaction kit and assembled either using Multalin [11] (http://www.prodes.toulouse.inra.fr/multalin/multalin.html) or manually by eye. Selected sequences have been deposited in GenBank with accession numbers EU251493–EU251501.

## Results

### PCR amplifications and gel electrophoresis results at STR loci

In this work parasite genotypes were determined using six tRNA-linked polymorphic markers containing STRs [7]. For the 16 stool samples from Bangladesh we used nested PCR, but nevertheless PCR still was not successful for all STR loci in all samples. In most cases, it was the amplification of the stool-derived DNA that was unsuccessful, while the corresponding liver abscess DNA amplified well. Locus S-Q showed multiple band patterns for most of the stool samples for unknown reasons and was excluded from further analysis. Among the other loci, the amplifications for D-A, A-L and S^TGA^-D were most successful, while those for R-R and N-K2 were least successful. These differences may reflect the copy number of the targets [12]. The results at these 5 loci for all sample pairs are shown in Table 1.

**Table 1:** PCR patterns with DNA samples from liver abscess and stool specimens of the same ALA patients.

Patient numbers	Patient ID	*PCR patterns					
		A-L	D-A	N-K2	R-R	S^TGA^-D	Overall
1	BAN-1	s	s	d	s	s	d
2	BAN-2	#	s	#	d?	d	d
3	BAN-3	d	d	#	d	d	d
4	BAN-4	d	d? (seq)	-	-	d	d
5	BAN-5	d (seq)	d	-	-	s (seq)	d
6	BAN-6	d	d	-	-	d (seq)	d
7	BAN-7	d	s (seq)	d	-	d	d
8	BAN-8	d	d	-	-	d	d
9	BAN-9	d	s	-	-	d	d
10	BAN-10	d	d (seq)	d	-	d	d
11	BAN-11	d?	d? (seq)	-	-	d	d
12	BAN-12	d? (seq)	s	#	s (seq)	#	d?
13	BAN-13	s (seq)	d	d (seq)	s	s (seq)	d
14	BAN-14	s	d	#	d? (seq)	#	d
15	BAN-15	s	#	#	d? (seq)	#	d?
16	BAN-16	s	s	#	s	d (seq)	d
17	Italy	s (seq)	s (seq)	s (seq)	s (seq)	d (seq)	d
18	USA	s (seq)	s (seq)	d (seq)	s (seq)	s (seq)	d

**Figure 1:**
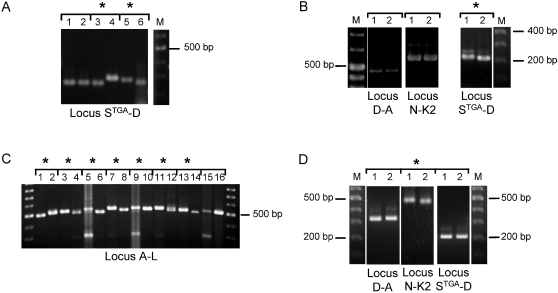
PCR product size polymorphism in paired samples. PCR product size polymorphism at selected STR loci using DNA samples from liver abscess (odd numbered lanes) and stool specimens (even numbered lanes) from the following amebic liver abscess patients: A. BAN-1 (1 & 2), BAN-2 (3 & 4) and BAN-3 (5 & 6). B. The Italian amebic liver abscess patient. C. BAN-4 (1 & 2), BAN-5 (3 & 4), BAN-6 (5 & 6), BAN-7 (7 & 8), BAN-8 (9 & 10), BAN-9 (11 & 12), BAN-10 (13 & 14) and BAN-11 (15 & 16). D. The USA amebic liver abscess patient. Asterisks indicate a PCR product size difference in the paired samples. A more complete set of PCR products can be seen in Figure S3.

*:  = underlined are those shown in Figure 1; # = did not amplify from stool DNA; ‘-’ = negative for both samples; s = same sized product; d = different sized product; d? = difference was unclear; (seq) = these products have been sequenced.

**Figure 2:**
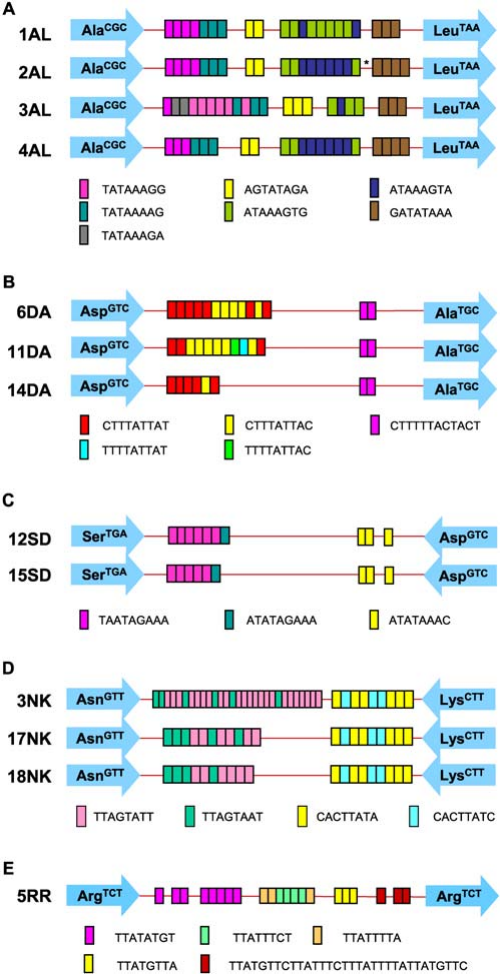
Sequence types of PCR products from paired samples. Schematic representations of (A) locus A-L, (B) locus D-A, (C) locus S^TGA^-D, (D) locus N-K2 and (E) locus R-R are given to illustrate the sequence type differences observed between paired samples. The asterisk indicates a single base insertion of A in the 2AL sequence. Each arrow represents a specific tRNA gene (shown inside the arrow) and colored boxes represent the STRs. The sequence types in loci N-K2, R-R, and S^TGA^-D were according to reference 13 (EF427346-EF427363, EF421375-EF421386 and EF421387-EF421403, respectively), and locus D-A sequence types were according to Clark, Ali and Haque (EU251498–EU251501 and unpublished). 5 sequence types have been detected to date in locus A-L (EU251493–EU251497).

For the majority of samples, gel electrophoresis showed that the overall patterns for stool and liver abscess sample pairs were different (Figure 1, Table 1). The only exceptions to this were sample pairs BAN-12 and BAN-15 (Figure S3). However, sequencing revealed that PCR products apparently of the same size were in fact distinct between these sample pairs (Table 2, Figure 2A and Figure S1B). The number of loci showing differences varied. Among Bangladeshi sample pairs, between 1 and 4 loci differed (Table 1). The Italian sample pair were identical for 5 loci, but showed a small size difference in locus S^TGA^-D (Figure 1B). Likewise, samples from the USA patient were also identical at 5 loci, but showed a small size difference in locus N-K2 (Figure 1D).

**Table 2:** Comparison between sequence types in liver abscess versus stool DNA from ALA patients.

ID	STR locus	Stool	Liver Abscess
BAN-5	A-L	1AL	2AL
BAN-12	A-L	3AL	4AL
BAN-13	A-L	4AL	4AL
Italy	A-L	2AL	2AL
USA	A-L	5AL	5AL
BAN-4	D-A	11DA	6DA
BAN-7	D-A	6DA	6DA
BAN-10	D-A	11DA	14DA
BAN-11	D-A	11DA	6DA
Italy	D-A	5DA	5DA
USA	D-A	6DA	6DA
BAN-13	N-K2	3NK	18NK
Italy	N-K2	11NK	11NK
USA	N-K2	18NK	17NK
BAN-12	R-R	5RR	5RR
BAN-14	R-R	5RR*	5RR*
BAN-15	R-R	5RR*	5RR*
Italy	R-R	6RR	6RR
USA	R-R	5RR	5RR
BAN-5	S^TGA^-D	12SD	12SD
BAN-6	S^TGA^-D	12SD	15SD
BAN-13	S^TGA^-D	12SD	12SD
BAN-16	S^TGA^-D	15SD	12SD
Italy	S^TGA^-D	12SD	15SD
USA	S^TGA^-D	15SD	15SD

*:  = Although both stool and liver abscess derived DNA showed identical STR patterns, there were point mutations detected outside of the STRs (shown in Figures S1A and S1B), and the stool and liver abscess derived sequences therefore differ from each other.

### Sequence results of PCR amplified products

Fifteen pairs of PCR products from the Bangladeshi samples were selected for sequencing. These included 6 pairs of products that were clearly different in size, 5 pairs that appeared to be the same size and 4 pair for which the size difference was ambiguous (an example of this shown in Figure S3G, lanes 1 and 2). All 6 loci were sequenced for both the Italian and USA cases. Sequence results confirmed all the gel observations, and in addition clearly revealed that the 4 pairs of Bangladeshi samples where the size differences were uncertain were indeed different in sequence (Table 2): BAN-12 and BAN-4 showed differences in number and arrangements of STRs in locus A-L and locus D-A, respectively (Figures 2A and 2B), while both BAN-14 and BAN-15 showed point mutations in their locus R-R sequences (see positions 312 and 349 in Figures S1A and S1B).

A striking finding was that for two pairs of samples (BAN-4 and BAN-11), the patterns of sequence difference at locus D-A were identical between the two liver abscess and stool samples (Table 2). In other words BAN-4^L^ and BAN-11^L^ were identical (sequence type 11DA) as were BAN-4^S^ and BAN-11^S^ (sequence type 6DA) [13]. Similarly, at locus S^TGA^-D, BAN-6^L^ and Italy^L^ were identical (sequence type 15SD) as were BAN-6^S^ and Italy^S^ (12SD) (Table 2). However, while the other loci in the Italian samples were identical between stool and liver abscess samples, this was not true for BAN-6 where loci D-A and A-L also differed (Table 1). The stool and all extra-intestinal samples from the USA patient showed identical STR patterns except for locus N-K2 where the extra-intestinal samples showed one extra 8 base pair repeat in an STR block compared to the stool sample (Table 2; Figure 2D).

In addition to STR variation, we also observed some single nucleotide polymorphisms (SNPs) among clones derived from the same DNA sample (some of these are shown in Figures S1A and S1B). These SNPs were present even though a high fidelity DNA polymerase was used for amplification prior to cloning. We have observed SNPs in different copies of STR loci from the same *E. histolytica* isolate previously (IKM Ali, unpublished data), and SNPs can be detected between reads in the HM-1:IMSS genome sequence where no PCR amplification was involved. Therefore it is likely that the origin of most SNPs is sequence variation among individual copies of loci within the same isolate. Direct sequencing of PCR products gives the ‘consensus’ sequence for the units, so any SNP differences would be obscured unless present in a majority of the copies. As an illustration of this, we observed a single base insertion/deletion difference in locus A-L between the paired samples from BAN-5 (Figure 2A; sequence type 2AL) and in locus R-R for pairs BAN-14 and BAN-15 (see below).

The possibility that the ‘liver abscess’ sequence is present in the intestinal population could be investigated in a few cases. Direct sequencing of PCR products from stool DNA at locus S^TGA^-D for the Italian case showed the presence of the ‘liver abscess’ version of the sequence in the form of minor peaks approximately 10% of the height of the consensus sequence peaks (Figure S2A). These minor peaks start at exactly the position expected for a sequence one STR copy shorter and can be clearly read. Likewise in the traces for the liver abscess sequence, minor peaks corresponding the intestinal consensus sequence can also be seen as minor peaks (approximately 5% of the consensus height) that extend the trace by 9 bases, the length of the relevant STR (Figure S2B). This is not a unique observation. The single base indel differentiating the stool and liver abscess R-R sequences in BAN-14 and BAN-15 produces a similar effect. In the stool DNA trace, a ‘shadow’ sequence is visible shifted one base to the right starting at exactly the point of the indel and continuing to the end of the product (Figure S2C). However there is no clear evidence for the alternative base at the position of the A/T transversion a few bases further away.

## Discussion

In *E. histolytica* there have been no reports comparing the genotypes of amebae identified in stool with those in the liver abscess pus of the same patients. However, in *Leishmania*, it was demonstrated that isolates from mucosal and cutaneous lesions of the same patients were genetically distinct and those authors suggested that a subpopulation of the parasite had reached the mucosal tissue from the original cutaneous lesion site [14]. We therefore were interested in investigating whether a similar phenomenon could be detected when comparing invasive and intestinal *E. histolytica*. In order to detect the genetic differences we used 6 highly polymorphic loci in *E. histolytica*. These loci are not likely to be directly involved in virulence, but they may be acting as surrogate markers and be physically linked to loci that are having a direct effect on the outcome of infection. Using this genotyping method, we have previously shown that the range of *E. histolytica* genotypes in liver abscess samples is different from that detected in intestinal samples in the Bangladeshi population [6],[8]. One limitation of our previous genotype comparisons was that they were conducted on samples from 3 separate clinical groups: asymptomatic, diarrhea/dysentery, and liver abscess; the first two groups provided stool samples, while the liver abscess patients gave only pus samples.

In 2004, while developing the genotyping methodology, we received two DNA samples from Prof. Egbert Tannich (Bernhard Nocht Institute, Hamburg) – one extracted from liver abscess pus from a woman who had never travelled outside of Europe and the other extracted from a stool sample provided by her husband. Typing showed the gel electrophoresis patterns for PCR products of the two samples to be distinct. This result inspired the present study, in which 18 pairs of *E. histolytica* DNA samples derived from stool and liver abscess pus of the same patient were collected. The samples were analysed by PCR genotype and sequencing to determine whether differences could be detected between amebae that had metastasized to liver tissue and those that remained in the intestine. The results we found were dramatic: the intestinal amebae were distinct from those in the liver abscess in all patients investigated, from Bangladesh, Italy and the USA. This observation based on sequencing of tRNA-linked STR loci was also supported by nested PCR of the widely studied polymorphic serine-rich *E. histolytica* protein (SREHP) gene. This gene encodes an immunodominant surface antigen containing tandem repeats of related dodeca- and octa-peptides [8],[15],[16],[17],[18]. Analysis of SREHP in three of the sample pairs from Bangladesh revealed that the intestinal and liver abscess amebae were indeed different from each other at this locus also (data not shown).

We believe that there are three possible explanations for these findings. Firstly, ALA may occur many months after the initial infection. Due to the time elapsed the original intestinal infection may have been cleared [19] and the amebae in our stool samples could be the result of a second infection with a distinct genotype. Genetic differences would then be expected due to the extensive diversity that exists even in geographically localized populations [6]. While a second infection is a reasonable possibility in an endemic country like Bangladesh, and could indeed be true in some of our cases, it seems extremely unlikely to be a realistic explanation for the cases from Italy and the USA where *E. histolytica* infection is rare.

Secondly, the original intestinal infection may have contained multiple genotypes, of which only one migrated to the liver via the bloodstream to cause the liver abscess (i.e. a “genetic bottleneck”). There are two different situations that could lead to multiple genotypes. The first is a ‘double infection’ scenario, where 2 or more unrelated genotypes are present, either through separate infections or exposure to a mixed source. The second scenario is a ‘heterogeneous infection’ of 2 or more related genotypes, most likely recently derived from a single source. The latter is more in line with what we see in the USA and Italian cases, where the stool and ALA pus genotypes were very similar - matching at 5 out of 6 loci sequenced. It would be unexpected to find infection with two such closely related genotypes by chance, given the diversity that exists in nature [8],[15],[16],[20]. In the Bangladeshi cases paired samples often differed at more loci than they shared, which is more in line with the double infection scenario. In both scenarios, however, only a minor genotype must have reached the liver.

Finally, there may be DNA reorganisation or recombination events taking place when the amebae migrate from the intestine to the liver. The STR loci at which the differences are being detected exist in multiple copies within a larger tandem array [12]. Such chromosomal structures are generally less stable than single-copy sequences [21]. The increase/decrease in STR number observed suggests a recombination event and tRNA genes are known to be ‘hotspots’ for recombination [22]. Although only one locus was detected as undergoing change in the Italian and USA paired samples, it should be remembered that only 6 unlinked STR loci were studied and there are 25 distinct tRNA gene arrays in the *E. histolytica* genome, each of which can have from 1 to 5 STRs. Even though only one locus was observed to change, recombination may well be much more extensive and widespread in the genome. It is also likely that in some future sample pairs no differences will be detected using these loci but that changes are nevertheless occurring elsewhere in the genome.

Interpreting the observations of ‘liver abscess’ sequences in stool DNA samples and vice versa is not straightforward. They could indicate the presence of a heterogeneous infection and the difference would then be the result of a genetic bottleneck through which a minor population of cells passed that happened to have indels involving STRs or single bases. It then becomes a problem to explain why in every case only a minor variant ended up in the liver, while the major genotype remained in the intestine. Alternatively, the observations could be the result of expansion of a variant array unit to replace the predominant version present in the intestinal cell population, implying radical recombination/reorganization of the array. Distinguishing between the two alternatives depends on whether all the cells in the intestinal infection would produce the same sequence trace or whether the relative proportions of the two sequence variants differs among cells. It is impossible to know which is the case.

Obtaining material for further studies of this type is not straightforward. Reports on the proportion of amebic liver abscess patients who harbor concomitant intestinal *E. histolytica* infections vary, with values ranging from less than 10% [23] to as high as 70% of patients [24]. Additionally, in most countries abscess drainage is not performed unless imminent rupture is anticipated, restricting the availability of ALA pus for study although to the benefit of the patient. However, in a study by Haque et al [9], lectin antigens were detected in the serum of 32 out of 98 ALA patients in Bangladesh and, in a more recent study by Parija and Khairnar [25], *E. histolytica* DNA was detected in the urine of 21 out of 53 ALA patients in India. These indicate that it might be possible to amplify *E. histolytica* DNA from the blood and/or urine of the ALA patients, removing the need for pus samples. It would then be possible to investigate intestinal and invasive ameba genotypes in many more ALA patients.

In order to determine if the differences between intestinal and liver isolates of *E. histolytica* in the same patient represent a genetic bottleneck effect or mutation will require additional experimentation. The ability to produce intestinal infections in mice using cloned isolates of *E. histolytica* [26] may allow the testing of our hypotheses. Nevertheless, our observations to date give an insight into why in our previous work the ALA genotypes differed from those in the intestine [6] and reinforce the likelihood that the ameba genotype is a significant player in the outcome of infection with *E. histolytica.*

## Supporting Information

Figure S1 Alignment of cloned sequences in locus R-R. (A) Three cloned sequences of locus R-R each from liver abscess (BAN-14LA) and stool (BAN-14ST) strains were aligned. At position 312, all 3 clones from liver abscess strain showed a ‘T’ insertion compared to that in all 3 clones from intestinal strain. At position 349, a consistent ‘T’ to ‘A’ conversion was observed between liver abscess and intestinal strains. (B) Three cloned sequences of locus R-R from liver abscess (BAN-15LA) and 4 cloned sequences from stool (BAN-15ST) strains were aligned. At position 312, all 3 clones from liver abscess strain showed a ‘T’ insertion compared to that in all 4 clones from intestinal strain. At position 349, a consistent ‘T’ to ‘A’ conversion was observed between liver abscess and intestinal strains.
(4.78 MB TIF)

Figure S2 Sequencing traces indicating the presence of alternative sequence variants. A. Reverse complement of locus S^TGA^-D from the stool DNA of the Italian amebic liver abscess patient. The sequence in black text under the trace is that of the minor peaks that can be seen, and is consistent with a variant that is shorter by one STR. B. Reverse complement of locus S^TGA^-D from the liver abscess of the Italian patient. The sequence in black text under the trace is that of a minor product and is consistent with a variant that is longer by one 9 base STR. C. Traces showing effect of indel. The upper traces is the sequence of locus R-R from the stool DNA and the lower trace that from the ALA DNA of BAN-14. The location of the indel is indicated by the 6T/7T notation and the box highlights the identity of the minor peaks in the upper trace with the sequence of the lower trace. The arrows indicate the position of the transversion that differentiates the two sequences (also seen in Figure S1).
(2.48 MB TIF)

Figure S3 PCR product size polymorphism in paired samples. Parts E, F, K and L are the same as those illustrated in Figure 1 of the main article. (A–E) PCR product size polymorphism at 5 STR loci using DNA samples from liver abscess (odd numbered lanes) and stool specimens (even numbered lanes) of 3 amebic liver abscess patients: BAN-1 (1 & 2), BAN-2 (3 & 4) and BAN-3 (5 & 6). (F–H) Polymorphism at 3 STR loci using DNA samples from liver abscess (odd numbered lanes) and stool (even numbered lanes) specimens of 8 amebic liver abscess patients: BAN-4 (1 & 2), BAN-5 (3 & 4), BAN-6 (5 & 6), BAN-7 (7 & 8), BAN-8 (9 & 10), BAN-9 (11 & 12), BAN-10 (13 & 14) and BAN-11 (15 & 16). (I–J) Representative PCR product size polymorphism using DNA samples from liver abscess (odd numbered lanes) and stool specimens (even numbered lanes) of 5 amebic liver abscess patients from Bangladesh: BAN-12 (1 & 2), BAN-13 (3 & 4), BAN-14 (5 & 6), BAN-15 (7 & 8), and BAN-16 (9 & 10). (K) PCR product size polymorphism at 3 STR loci using DNA samples from liver abscess (LA) and stool (ST) specimens of the Italian amebic liver abscess patient. (L) PCR product size polymorphism at 3 STR loci using DNA samples from liver abscess (LA) and stool (ST) specimens of the USA amebic liver abscess patient. Asterisks indicate a PCR product size difference in the paired samples.
(3.02 MB TIF)
