# Correction: Tissue Invasion by *Entamoeba histolytica:* Evidence of Genetic Selection and/or DNA Reorganization Events in Organ Tropism

**DOI:** 10.1371/annotation/6fb9dd82-4678-48cb-a3b4-9856fdbe58e5

**Published:** 2008-06-02

**Authors:** Ibne Karim M. Ali, Shahram Solaymani-Mohammadi, Jasmine Akhter, Shantanu Roy, Chiara Gorrini, Adriana Calderaro, Sarah K. Parker, Rashidul Haque, William A. Petri, C. Graham Clark

There was an error in Figure 2A and in the legend to Figure 2. Please see the correct Figure 2 here.

The second sentence of the legend for Figure 2 should read: "The asterisk indicates a single base insertion of T in the 2AL sequence."

**Figure 2:**
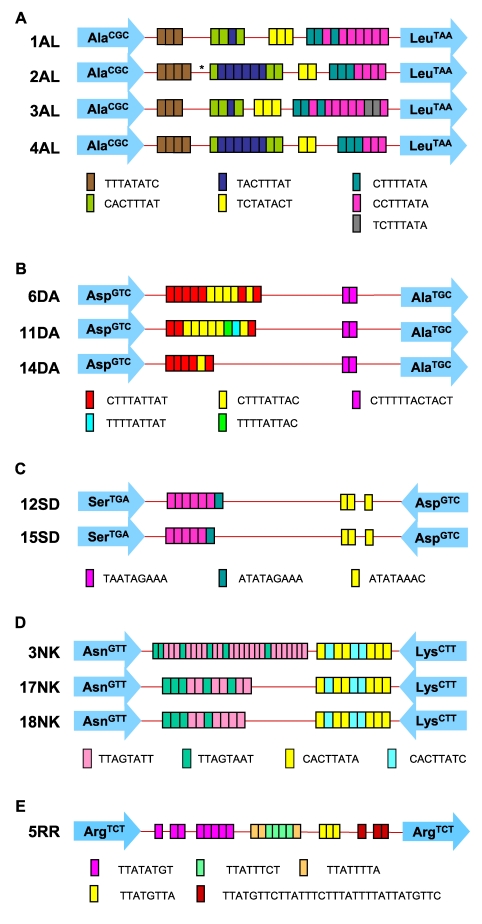
Sequence types of PCR products from paired samples. Schematic representations of (A) locus A-L, (B) locus D-A, (C) locus S^TGA^-D, (D) locus N-K2 and (E) locus R-R are given to illustrate the sequence type differences observed between paired samples. The asterisk indicates a single base insertion of T in the 2AL sequence. Each arrow represents a specific tRNA gene (shown inside the arrow) and colored boxes represent the STRs. The sequence types in loci N-K2, R-R, and S^TGA^-D were according to reference 13 (EF427346-EF427363, EF421375-EF421386 and EF421387-EF421403, respectively), and locus D-A sequence types were according to Clark, Ali and Haque (EU251498–EU251501 and unpublished). 5 sequence types have been detected to date in locus A-L (EU251493–EU251497).

