# Supplementary figures and images for: Tissue Invasion by Entamoeba histolytica: Evidence of Genetic Selection and/or DNA Reorganization Events in Organ Tropism
Source: PLoS Negl Trop Dis. 2008 Apr 9;2(4):e219. doi: 10.1371/journal.pntd.0000219 (PMC2274956; doi:10.1371/journal.pntd.0000219)

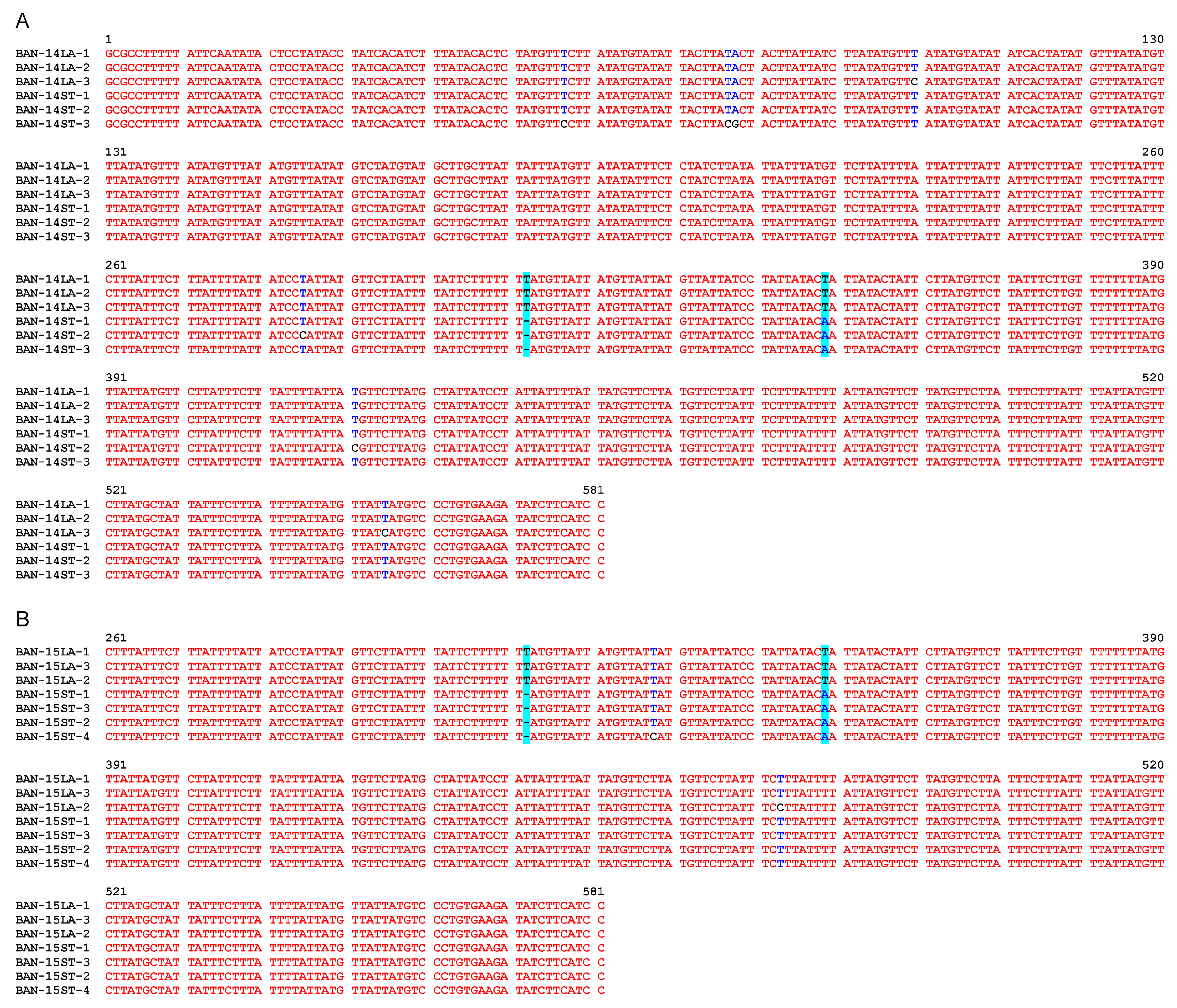

Supplement: Figure S1 — Alignment of cloned sequences in locus R-R. (A) Three cloned sequences of locus R-R each from liver abscess (BAN-14LA) and stool (BAN-14ST) strains were aligned. At position 312, all 3 clones from liver abscess strain showed a ‘T’ insertion compared to that in all 3 clones from intestinal strain. At position 349, a consistent ‘T’ to ‘A’ conversion was observed between liver abscess and intestinal strains. (B) Three cloned sequences of locus R-R from liver abscess (BAN-15LA) and 4 cloned sequences from stool (BAN-15ST) strains were aligned. At position 312, all 3 clones from liver abscess strain showed a ‘T’ insertion compared to that in all 4 clones from intestinal strain. At position 349, a consistent ‘T’ to ‘A’ conversion was observed between liver abscess and intestinal strains. (4.78 MB TIF) [file pntd.0000219.s001.tif]

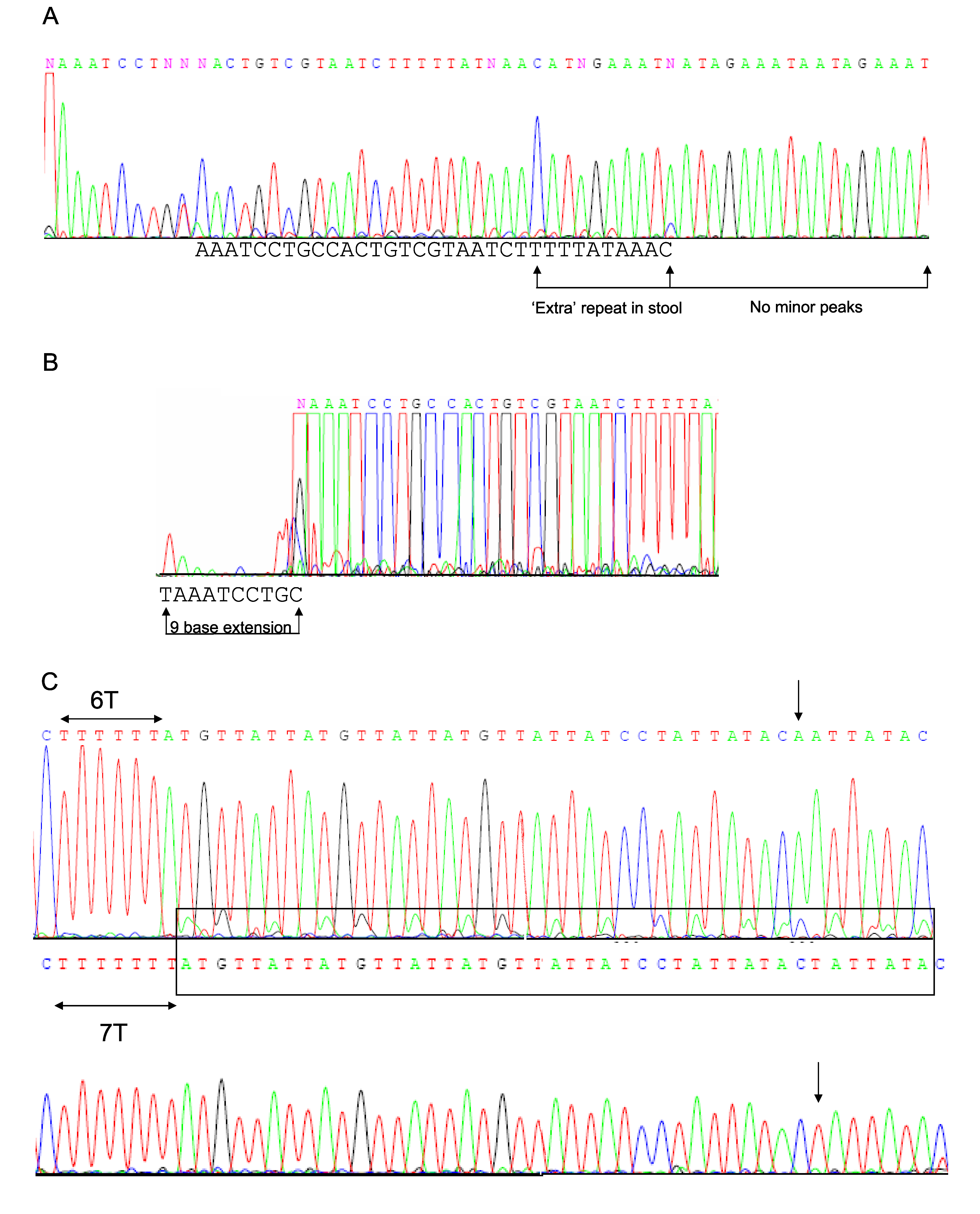

Supplement: Figure S2 — Sequencing traces indicating the presence of alternative sequence variants. A. Reverse complement of locus STGA-D from the stool DNA of the Italian amebic liver abscess patient. The sequence in black text under the trace is that of the minor peaks that can be seen, and is consistent with a variant that is shorter by one STR. B. Reverse complement of locus STGA-D from the liver abscess of the Italian patient. The sequence in black text under the trace is that of a minor product and is consistent with a variant that is longer by one 9 base STR. C. Traces showing effect of indel. The upper traces is the sequence of locus R-R from the stool DNA and the lower trace that from the ALA DNA of BAN-14. The location of the indel is indicated by the 6T/7T notation and the box highlights the identity of the minor peaks in the upper trace with the sequence of the lower trace. The arrows indicate the position of the transversion that differentiates the two sequences (also seen in Figure S1). (2.48 MB TIF) [file pntd.0000219.s002.tif]

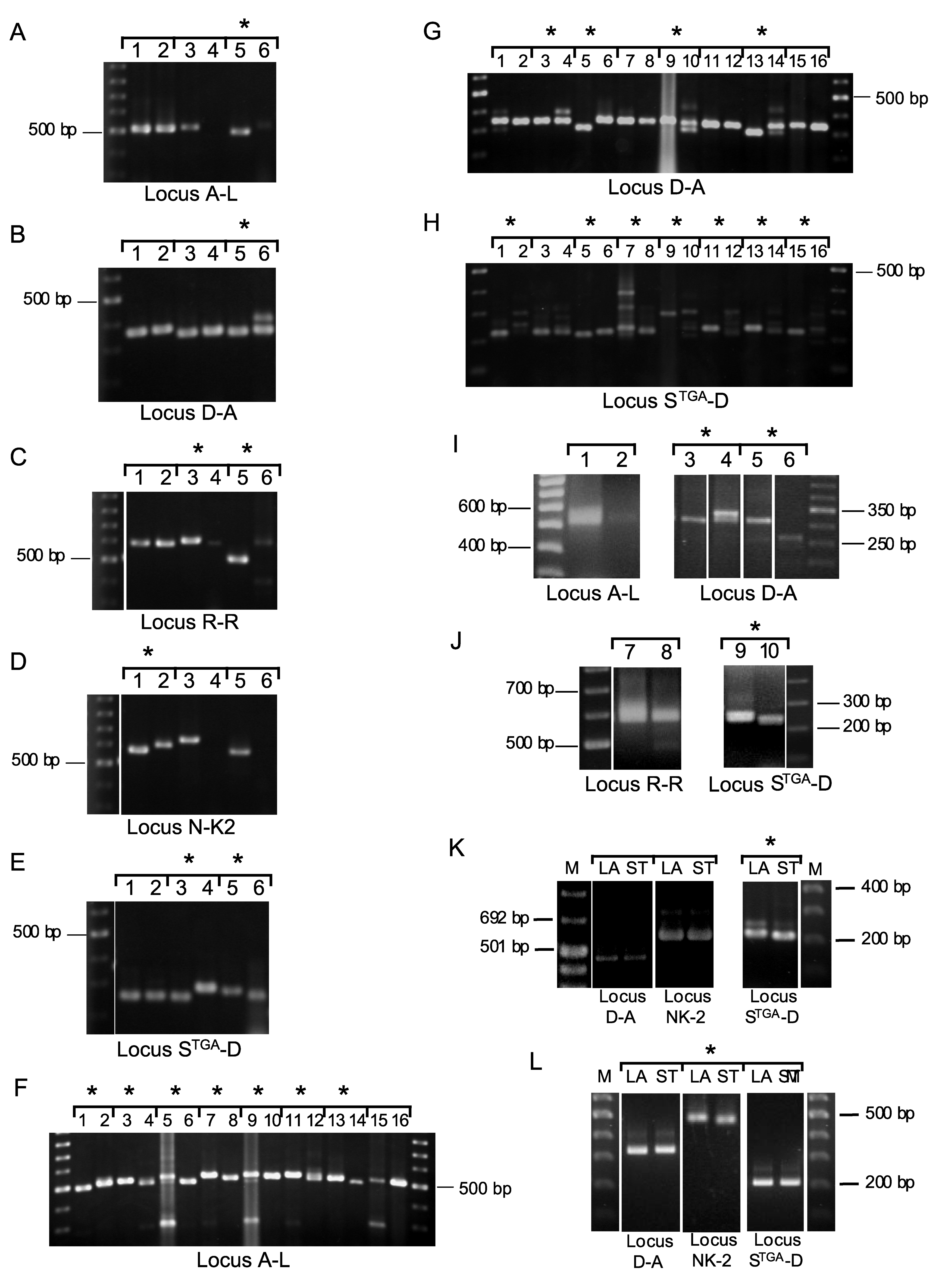

Supplement: Figure S3 — PCR product size polymorphism in paired samples. Parts E, F, K and L are the same as those illustrated in Figure 1 of the main article. (A–E) PCR product size polymorphism at 5 STR loci using DNA samples from liver abscess (odd numbered lanes) and stool specimens (even numbered lanes) of 3 amebic liver abscess patients: BAN-1 (1 & 2), BAN-2 (3 & 4) and BAN-3 (5 & 6). (F–H) Polymorphism at 3 STR loci using DNA samples from liver abscess (odd numbered lanes) and stool (even numbered lanes) specimens of 8 amebic liver abscess patients: BAN-4 (1 & 2), BAN-5 (3 & 4), BAN-6 (5 & 6), BAN-7 (7 & 8), BAN-8 (9 & 10), BAN-9 (11 & 12), BAN-10 (13 & 14) and BAN-11 (15 & 16). (I–J) Representative PCR product size polymorphism using DNA samples from liver abscess (odd numbered lanes) and stool specimens (even numbered lanes) of 5 amebic liver abscess patients from Bangladesh: BAN-12 (1 & 2), BAN-13 (3 & 4), BAN-14 (5 & 6), BAN-15 (7 & 8), and BAN-16 (9 & 10). (K) PCR product size polymorphism at 3 STR loci using DNA samples from liver abscess (LA) and stool (ST) specimens of the Italian amebic liver abscess patient. (L) PCR product size polymorphism at 3 STR loci using DNA samples from liver abscess (LA) and stool (ST) specimens of the USA amebic liver abscess patient. Asterisks indicate a PCR product size difference in the paired samples. (3.02 MB TIF) [file pntd.0000219.s003.tif]
